# Usage of DNA Fingerprinting Technology to Check Sample Error and Contamination in Molecular Laboratories

**DOI:** 10.3390/cimb44110375

**Published:** 2022-11-08

**Authors:** Dahui Qin, Mark Forster, Shital M. Gandhi, Ratilal Akabari, Zhong Zheng, Janella Lal, Katherine Lovinger

**Affiliations:** 1 Molecular Diagnostic Laboratory, Moffitt Cancer Center, Tampa, FL 33612, USA; 2 Molecular Diagnostic Laboratory, State University of New York (SUNY), Upstate Medical University, Syracuse, NY 13210, USA; 3 Molecular Diagnostic Laboratory, Emory University Hospital, Atlanta, GA 30322, USA; 4 Orlando Health (ORMC’s) Molecular Laboratory, Orlando, FL 32806, USA

**Keywords:** quality control, sample switch, sample contamination, sample error, DNA fingerprint

## Abstract

Sample identification error is a severe medical error in clinical molecular diagnostic laboratories, which can lead to reporting the wrong results for the patient involved. Sample contamination can also lead to incorrect test reports. Avoiding sample identification error and sample contamination could be life-saving. Sample switch and sample contamination could happen on laboratory bench works, especially when pipetting into multi-well plates. It is difficult to realize such errors during laboratory bench work. Laboratory staff may not be aware of such an error when it happens. DNA fingerprinting technology can be used to determine sample identity and subsequently identify sample switch and sample contamination in the laboratory. Our laboratory has explored the usage of this technology in our quality control process and successfully established that DNA fingerprinting can be used to monitor sample switch and sample contamination in next-generation sequencing and BCR/ABL1 real-time PCR bench work.

## 1. Introduction

Quality assurance (QA) is critical in clinical molecular diagnostic laboratories. One of the goals is to avoid sample switch and sample contamination. A pipetting error can cause sample switch or sample contamination, which can lead to reporting the wrong results. Such pipetting errors are difficult to identify when they happen during laboratory bench work. An effective QA procedure to monitor such pipetting errors can help improve laboratory QA. Deoxyribonucleic acid (DNA) fingerprinting is a powerful tool in determining sample identity and it has been used in many laboratories for post-transplant chimerism testing and forensic testing. This method uses short tandem repeats (STR) in the human genome as markers of an individual’s DNA fingerprints. The STR markers are usually polymorphic in the human genome and are often heterozygous among individuals, which provides sufficient diversity so that no individuals, except identical twins, will have the same DNA fingerprints when 16 markers are used [1,2,3,4,5,6]. This tool could be potentially used in monitoring accidental sample switch and sample contamination. Current DNA fingerprinting assays are very sensitive. These assays only need a trace amount of DNA to determine sample identity. A molecular diagnostic laboratory may carry out different molecular assays. Most of the assays involve multiple steps of sample processing, involving target amplification and cleaning, etc. Some assays use ribonucleic acid (RNA) as interrogating targets. However, regardless of these factors, it is still possible that a trace amount of DNA will survive different steps of different sample processing. Such trace amounts of DNA could still be detected by DNA fingerprint assay and DNA fingerprinting could therefore be used to detect sample identification (ID) error or sample contamination. The trace amount of DNA in the leftover samples offers us an opportunity to retrospectively check the sample DNA fingerprints, which reveal the real sample ID. In a previous piece of work, our lab has used this technology to detect sample ID error in gene rearrangement assay bench work [7]. Now, our laboratory has successfully established that DNA fingerprinting can be used in monitoring possible sample switch and sample contamination in-next generation sequencing and BCR/ABL1 real-time polymerase chain reaction (PCR) laboratory bench work.

## 2. Materials and Methods

DNA fingerprinting test: DNA fingerprinting was performed using Promega PowerPlex assay [8] by addition of 1 µL of the library mixtures from next-generation sequencing (NGS) preparations or 1 µL of the reaction mixtures from BCR/ABL1 assay preparations directly into 11.5 µL of the Promega PowerPlex^®^ 16 HS Amplification PCR admixture. These samples then went through a round of PCR amplification (Promega, 2800 Woods Hollow Road, Madison, WI, USA, per manufacturer’s protocol) on the C1000 Touch thermal cycler (Bio-rad, 1000 Alfred Nobel Drive, Hercules, CA, USA). After the amplification, 1 µL of the product was used for fragment analysis. The fragment analysis was performed on AB 3130xL. Data are analyzed using GeneMapper^®^ v4.1 software (Life Technologies, 3175 Staley Road, Grand Island, NY, USA) to obtain the DNA fingerprints. The DNA fingerprint was then compared with the original sample DNA fingerprint to check for any possible sample switch or sample contamination.

Sampling of NGS assay: our NGS assay uses an Illumina TruSight 54 gene panel, which uses a TruSeq Custom Amplicon kit for library preparation. The PCR is run in 27 cycles. There are multiple steps in NGS lab bench work, which include DNA extraction, library preparation, library quality control (QC) and quantitation, library concentration adjustment and NGS sequencing [9]. Sample switch and sample contamination could happen in any of these steps. To check this, 1 µL of library mixture from the last step of library preparation was used for testing. 

In the NGS library preparation, sample DNA was isolated using the QIAGEN Blood Mini Kit (Qiagen, 19300 Germantown Road Germantown, MD, USA). The libraries were prepared using a TruSeq™ Custom Amplicon Kit (Illumina, 5200 Illumina Way, San Diego, CA, USA). The library QC and quantitation was performed on a TapeStation (Bio-rad, 1000 Alfred Nobel Drive Hercules, CA, USA). From each sample, 50 ng of total DNA was used for library preparation. The libraries were sequenced on Illumina NextSeq. Thirty-two samples were tested.

Sampling of BCR/ABL1 real-time PCR assay: there are multiple steps in this real-time PCR assay, which include total nucleic acid extraction, reverse transcription and quantitative real-time PCR with 45 cycles [10,11]. For sample error checking purposes, 1 µL of quantitative real-time PCR mixture at the end step of this assay was used for testing.

In the BCR/ABL1 real-time PCR assay, the total nucleic acid was extracted from peripheral blood or bone marrow using the QIAGEN Blood Mini Kit (Qiagen, 19300 Germantown Road Germantown, MD 20874). The cDNA was synthesized using a high-capacity cDNA reverse transcription kit (Applied Biosystems Cat log #: 4368814). BCR/ABL1 qPCR was performed using an ABI Taqman gene expression master mix (Applied Biosystems, Cat log # 4304437). 

## 3. Results

### 3.1. DNA Fingerprinting Test on NGS Library

**Figure 1:**
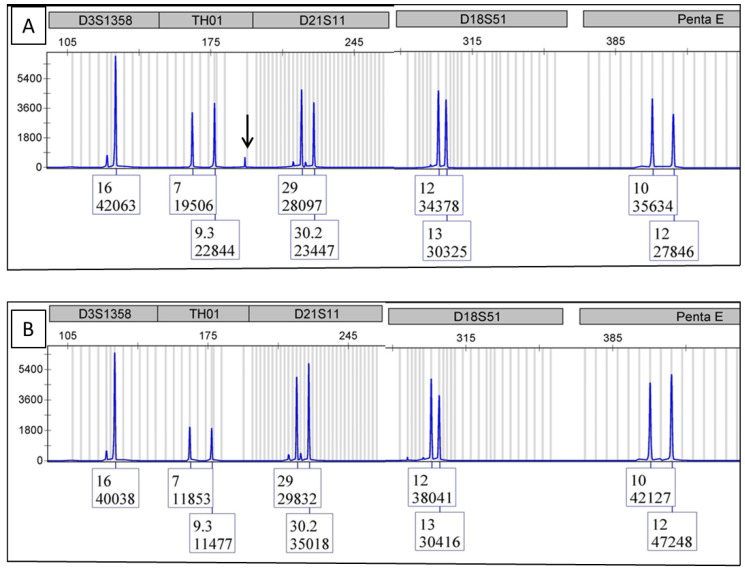
This figure consists of the electropherograms from multiple samples of the same category to protect the DNA fingerprint of any one sample. (**A**) is the DNA fingerprint from the NGS library. The arrow indicates a non-specific peak. (**B**) is the DNA fingerprint from the original sample.

DNA fingerprints were detected from the NGS library mixture at the last step of the library preparation, though there were occasional, small, non-specific peaks as noisy background (Figure 1A). Figure 1B shows the DNA fingerprint from the original sample DNA.

Comparative analysis of the PCR product fingerprint to the original DNA fingerprint (Figure 1B), which was run in parallel, showed matching allelic markers between the two electropherograms, confirming proper sample identification. An occasional, small, non-specific peak could be seen (Figure 1A, black arrow). However, it did not compromise the data comparison.

### 3.2. DNA Fingerprinting Test on the Samples in the BCR/ABL1 Real-Time Assay

**Figure 2:**
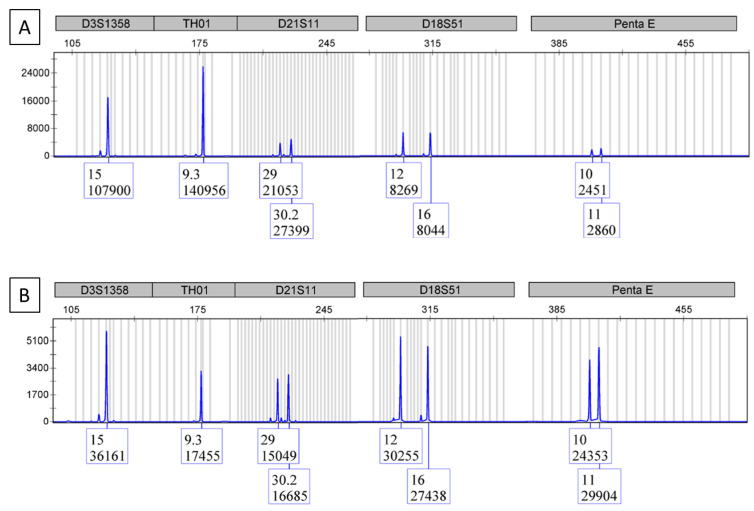
This figure consists of the electropherograms from multiple samples of the same category to protect the DNA fingerprint of any one sample. (**A**) is the DNA fingerprint from the BCR/ABL1 real-time PCR reaction mixture. (**B**) is the DNA fingerprint from the original sample.

Although the BCR/ABL1 test is designed to test RNA, total nucleic acid was extracted for testing, which includes genomic DNA. After reverse transcription and quantitative PCR, the PCR reaction mixture was tested using a DNA fingerprinting assay. The results show that DNA fingerprints can be detected (Figure 2A). The data show that different DNA fingerprint markers survived the real-time PCR process in different manners. In our case, the D3S1358 and TH01 markers survived better than the D21S11, D18S51 and Penta E (Figure 2A). However, it did not compromise the data comparison. The attempts of adding more BCR/ABL1 reaction mixture into the DNA fingerprint test resulted in more noisy background.

Comparative analysis of the PCR product fingerprint to the original DNA fingerprint (Figure 2B), which was run in parallel, showed matching allelic markers between the two electropherograms, thus confirming proper sample identification. 

## 4. Discussion

In a clinical molecular laboratory, a pipetting error can cause a switch in patient samples, which can lead to reporting the wrong results for the patient samples involved. Normally, pipetting errors are unintentional and difficult to realize during the pipetting process. Finding a way to detect the potential sample switch or sample contamination on the lab bench is important. DNA fingerprinting technology provides a possibility to achieve this goal in determining sample identification [1,2] and it could be potentially used in detecting accidental sample switch and sample contamination. DNA fingerprinting is a powerful technology. It has been widely used for human identification. The Federal Bureau of Investigation (FBI) in the United States has been using it to obtain human identification data for the Combined DNA Index System (CODIS) as a criminal justice DNA fingerprinting databases [12]. It is also used to obtain data for the European Standard Set (ESS) of DNA Database [13,14].

The DNA fingerprint markers are short nucleotide tandem repeats. Our results show that certain amounts of genomic DNA, which contains the short tandem repeats and flanking genomic DNA, survived the NGS library preparation process and could be amplified and detected in the library preparation. The DNA fingerprints of the library preparation can be compared with the DNA fingerprints of the original sample to check if there is any sample switch. Such comparisons can also be used to detect sample contamination. Our DNA fingerprinting assay consists of 16 short tandem repeats (STRs), which are also called markers. The newer assay could have 22 markers [15]. Each marker consists of two alleles, with one being from a biological father and the other being from a biological mother. At each marker, an individual could be either homozygous or heterozygous. When an individual is homozygous for a certain marker, the test result will show one peak for that marker. When an individual is heterozygous for a certain marker, the test result will show two peaks for that marker. If a test result shows more than two peaks for a certain marker, that means that the sample is from a mixture of more than one individual. If there is a sample contamination in a NGS library, the DNA fingerprint of the NGS library will show the DNA fingerprints from more than one individual.

Although occasional small non-specific peaks can be present in the DNA fingerprints from the NGS library preparation, it will not compromise the DNA fingerprint comparison. If all the DNA fingerprint markers from the NGS library match the markers from the original sample, we know there is no sample ID error even though there is a random, small, non-specific peak. 

We have encountered a scenario in an NGS run, where one polycythemia vera (PV) case was negative for JAK2 V617F and a mycosis fungoides (MF) case in the same run was positive for JAK2 V617F. Normally, PV cases carry a JAK2 V617F mutation, while MF cases usually do not have a JAK2 V617F mutation. Therefore, a sample switch was strongly suspected. We carried out a DNA fingerprint check on the library preparation of both samples. DNA fingerprinting results indicated that there was no sample switch. Further investigation found that there was a clerical error for ‘mycosis fungoides’. The diagnosis of this sample was initially labeled as ‘MF’, which was interpreted as ‘mycosis fungoides’. In fact, MF was used as the abbreviation of Myelofibrosis. Myelofibrosis is a disease, which is often positive for a JAK2 V617F mutation.

In the case of sample contamination, the occasional, small, non-specific peak will not compromise data comparison. If there is a sample contamination, one would expect the DNA fingerprint from the contaminating DNA to be seen in multiple markers. Our current DNA fingerprint test has 16 markers. As for how to define “multiple markers”, we believe it is prudent not to set up an arbitrary number without knowing how many informative markers there are between the contaminating DNA and the tested sample. In our practice, when a sample contamination is suspected, we will test all the samples in the same run, which will help to identify the source of the contamination and of course confirm the sample contamination.

DNA fingerprinting technology can also be used to check sample switch and sample contamination for BCR/ABL1 real-time PCR assay. Although the BCR/ABL1 test is designed to test RNA, total nucleic acid was extracted for testing, which includes genomic DNA. Our results show that a certain amount of genomic DNA survives the real-time PCR process and the DNA fingerprint markers can be detected in the end product of this assay and be used for sample ID checking. 

**Figure 3:**
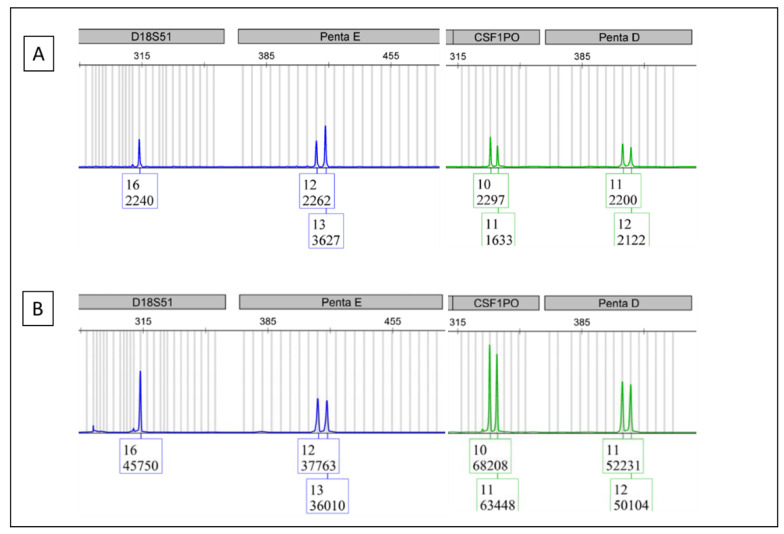
This figure consists of the electropherograms from multiple samples of the same category to protect the DNA fingerprint of any one sample. (**A**) is the DNA fingerprint from a common reagent. (**B**) is the DNA fingerprint from the BCR/ABL1 real-time PCR reaction mixture with a high BCR/ABL1 level.

Apart from checking samples, DNA fingerprinting technology can also be used in checking lab reagents for contamination. In fact, we have encountered a scenario where multiple samples from the patients in complete remission were noticed to have unexpected BCR/ABL1 positivity. In the QC process, DNA fingerprinting was used to test all the reagents used in this run. A common reagent used in the robotic nucleic acid (NA) extraction process tested positive (Figure 3A). This common reagent is not supposed to have any human DNA in it. Therefore, a positive result indicates that it is contaminated by human DNA. The real-time PCR mixtures of all samples from that run were tested. A patient’s sample with a high BCR/ABL1 level showed the same DNA fingerprint as that seen in the common reagent (Figure 3B) indicating that this sample contaminated the common reagent and went on contaminating the other samples, which led to false positivity in the patients in complete remission.

**Figure 4:**
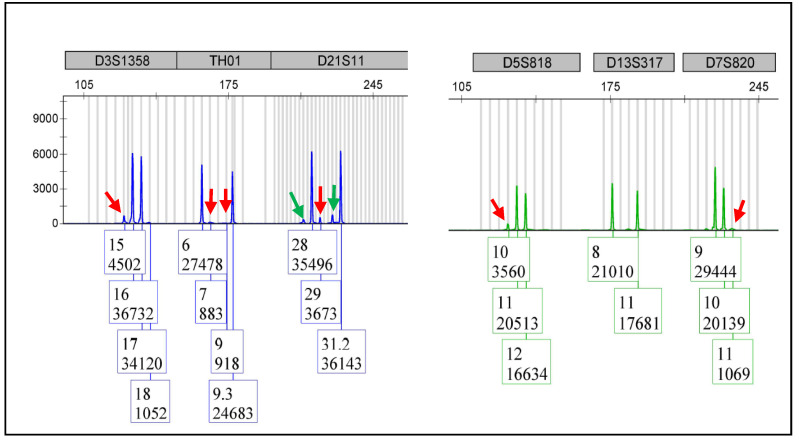
This figure consists of the electropherograms from multiple samples of the same category to protect the DNA fingerprint of any one sample. The DNA from two individuals were mixed with one of them contributing 5% of total DNA as a surrogate of contaminating DNA. Such ‘contaminating’ DNA was detected as indicated by the low peaks (red arrows). Please note that D13S317 is a non-informative marker for this mixture. The two low peaks of D21S11 (green arrows) can be considered as stutters.

DNA fingerprinting technology is a relatively sensitive technology. Many molecular diagnostic laboratories have been using it in engraftment tests for bone marrow transplant patients. In general, such technology can detect 5% of donor cells in a recipient. Figure 4 shows the data of a DNA mixture from two individuals, with one of them contributing 5% of the total DNA as a surrogate of ‘donor’ or ‘contaminating’ DNA. The data show that the STR markers of 5% of ‘contaminating’ DNA could be detected, though they were lower in some markers. However, in reality, when checking sample DNA contamination, the quality of the sample DNA varies. For some samples, the test sensitivity is 5%. For some other samples, the quality of DNA may be suboptimal, and the test sensitivity may deviate from 5%.

## 5. Conclusions

In short, DNA fingerprinting technology can be useful in detecting sample switch and sample contamination in both NGS and BCR/ABL1 assays. This method may potentially be a valuable technique used to monitor sample switch and sample contamination in other molecular assays.

## Data Availability

Not applicable.
